# An efficient RGB-UAV-based platform for field almond tree phenotyping: 3-D architecture and flowering traits

**DOI:** 10.1186/s13007-019-0547-0

**Published:** 2019-12-26

**Authors:** Francisca López-Granados, Jorge Torres-Sánchez, Francisco M. Jiménez-Brenes, Octavio Arquero, María Lovera, Ana I. de Castro

**Affiliations:** 1 Institute for Sustainable Agriculture IAS-CSIC, 14004 Córdoba, Spain; 2 Institute of Agricultural Research and Training (IFAPA-Alameda del Obispo), 14004 Córdoba, Spain

**Keywords:** Low-cost camera, Volume and 3D mapping, Colored point clouds, Object-based image analysis (OBIA), Remote sensing, Woody crop

## Abstract

**Background:**

Almond is an emerging crop due to the health benefits of almond consumption including nutritional, anti-inflammatory, and hypocholesterolaemia properties. Traditional almond producers were concentrated in California, Australia, and Mediterranean countries. However, almond is currently present in more than 50 countries due to breeding programs have modernized almond orchards by developing new varieties with improved traits related to late flowering (to reduce the risk of damage caused by late frosts) and tree architecture. Almond tree architecture and flowering are acquired and evaluated through intensive field labour for breeders. Flowering detection has traditionally been a very challenging objective. To our knowledge, there is no published information about monitoring of the tree flowering dynamics of a crop at the field scale by using color information from photogrammetric 3D point clouds and OBIA. As an alternative, a procedure based on the generation of colored photogrammetric point clouds using a low cost (RGB) camera on-board an unmanned aerial vehicle (UAV), and an semi-automatic object based image analysis (OBIA) algorithm was created for monitoring the flower density and flowering period of every almond tree in the framework of two almond phenotypic trials with different planting dates.

**Results:**

Our method was useful for detecting the phenotypic variability of every almond variety by mapping and quantifying every tree height and volume as well as the flowering dynamics and flower density. There was a high level of agreement among the tree height, flower density, and blooming calendar derived from our procedure on both fields with the ones created from on-ground measured data. Some of the almond varieties showed a significant linear fit between its crown volume and their yield.

**Conclusions:**

Our findings could help breeders and researchers to reduce the gap between phenomics and genomics by generating accurate almond tree information in an efficient, non-destructive, and inexpensive way. The method described is also useful for data mining to select the most promising accessions, making it possible to assess specific multi-criteria ranking varieties, which are one of the main tools for breeders.

## Background

Almond (*Prunus dulcis* (Mill.) D.A. Webb) is an emerging and valuable crop due to the health benefits of almond consumption, including nutritional, anti-inflammatory, and hypo-cholesterolemia properties [1], and to the wide range of applications of almond by-products, e.g., almond shells are burned as fuel, and almond hulls are used as livestock feed [2], which increases almond’s potential profitability. Almond producers were traditionally concentrated in California, Australia, and Mediterranean countries; however, almond is currently grown in more than 50 countries. Together with its health properties, other explanations for the expansion of almond growth are the favorable market conditions given by good almond prices and fruit deficiency in Europe (https://faostat.fao.org), and the necessity of increasing the sustainability and diversification of agricultural production in many areas of the world. An example of the last point is the Mediterranean Basin, where the olive monoculture can be complemented by almond [3]. In fact, almond is a useful alternative crop because its agricultural machinery can be shared by both kinds of orchards, since their harvest and cultural practices are carried out on different dates (olive is harvested in winter and almond in late summer).

Different almond tree ages and sizes can affect the yield of every cultivar in high-yielding breeding programs [4], and temperatures below ‒ 1 °C recorded in late frosts can cause cold damage during almond tree flowering and early fruit development [5]. For these reasons, the characterization of almond tree architecture and also of an appropriate flowering time are critical for breeders to develop new varieties adapted to cooler climates to achieve a stable and long-lasting high yield. Monitoring of flowering time is also one of the most important agronomic tasks in almond breeding, because in order to use cultivars for cross-pollination to achieve successful pollination, the flowering times of two varieties must coincide [6]. Consequently, a main objective for almond breeders has been to develop new genotypes with improved desirable traits related to tree architecture and high yield, which is directly related to reproduction and late flowering, thereby avoiding late frosts that negatively affect production in early flowering cultivars [7]. The main interest for breeders is based on the following:

(i) Tree architecture: Describes the tree form by defining the spatial organization of different tree structural components [8]. Tree geometry can influence the behavior of almond varieties by affecting the fruit-bearing habit, tree size and form, or light penetration and capture by the canopy [9]. The growth pattern and size of an almond variety are also important because of their influences on cultural techniques such as tree spacing, training, and pruning systems and their consequent effects on production level as well as crop production costs [10].
(ii) Traits related to reproduction: The aim is to ensure a high and stable yield. This is an important feature of varieties in the evaluation of the productive potential and its value as a commercial cultivar [7].
(iii) Flowering: As stated before, late blooming is a primary objective in almond breeding programs. Late flowering varieties are less likely to be affected by frost during flowering, which causes premature crop loss or decrease [11]. The creation of late and very late flowering varieties makes almond production in cold inland areas possible, where almond growing was impossible with the old almond early flowering varieties [12].

Gülcan [13] described and coded the standard almond descriptors for breeders including flowering, tree habits, tree vigor, branching intensity, and nut size, among others. All of these traits are evaluated through intensive field labor in phenotyping experiments to select the varieties with the most desirable traits. However, in some instances, not all trees or all features of a screening experimental field can be timely characterized and measured, often due to a lack of resources or workforce. Another problem related to the use of field measurements is that tree crown volume calculation is usually based on the use of volume equations of geometric models such as cones, ellipsoids, hemi-spheres, or ovoids [14]. This fact causes the estimations to be inaccurate because of the lack of fit of these geometric solids to the irregular tree crowns due to the branches and the more complex internal configuration. To solve these problems related to the lack of access to efficient phenotyping capabilities to scrutinize the quantitative traits related to varieties, efficient sensors and workflows that can map the geometric properties and phenological stages of trees growing in experimental orchards are needed [15].

In recent years, the use of Unmanned Aerial Vehicles (UAVs) has allowed the acquisition of images with high spatial resolution and overlap, which enables the generation of accurate 3D models using photogrammetry and structure from motion (SfM) techniques for different geomatic applications [16, 17]. One of the emerging applications of 3D models derived from UAV imagery using Digital Surface Models (DSMs) is their use in agriculture in woody crops for mapping the geometric traits of individual trees such as olives [18] and vineyards [19, 20]. However, volume tree monitoring using 3D point clouds rather than DSMs has become a relevant advancement because photogrammetric techniques from UAV images can yield a 3D point cloud similar to that produced by LiDAR (light detection and ranging) systems [21]. But also because the 3D point clouds allow the mapping of complex and highly variable structures, such as tree orchards in vineyards [19, 22] and lychee trees [23]. Recently, Torres-Sánchez et al. [24] accurately mapped almond tree height using 3D point clouds (R^2^ = 0.94 and RMSE = 0.39 m in comparison with field data). The high accuracy they achieved allowed them to generate 3D maps for the volume of every tree as well as the volume growth to design site-specific treatments (pruning, fungicides) adapted to the necessities of every tree according to its size. They concluded that point clouds outperform DSMs in 3D reconstruction of trees since a DSM only represents the upper part of the crown, while in a point cloud the lower part can also be depicted. Point clouds are real 3D models because they can store more than a single height value (Z) at each coordinate (X, Y), while DSMs are defined as 2.5 datasets as they only can represent a Z value at each 2D coordinate [25].

In terms of efficiency, the UAV platforms are considered efficient and inexpensive tools [26]. It has been argued that the cost of UAV imagery is lower than that of on-ground technologies based on depth cameras or LiDAR, and they can cover larger areas [27]. They reported that, although every technique provided reliable and similar results for volume calculation in vineyard, the cost of acquisition was always higher than that of aerial imagery. In addition, Rueda Ayala et al. [28] evaluated aerial and on-ground methods to characterize grass ley fields in terms of pasture biomass, concluding that UAV-based plant height and volume estimation offers major advantages over on-ground technologies. Namely, UAV can be properly classified as a non-destructive sampling method, while the on-ground methods are not fully non-destructive in the case of absence of pathways to walk through the field without damaging the crop.

Due to their affordability and efficiency in producing a large amount of high-quality geospatial data collection in a short period of time, UAVs and their associated 3D models are a powerful tool for field phenotyping studies to relieve the bottleneck by providing a rapid field evaluation of a large number of trials for crop breeding programs [26, 29]. Most of the research about the use of UAVs as sensing platforms for field-based phenotyping has been focused on herbaceous crops such as wheat [30, 31], sorghum [32], cotton [33], or maize [34]. Less research has been addressed their use for woody crop phenotyping, which has been developed in olive for the measurement of tree height and crown diameter [35, 36]. Yang et al. [26] reviewed the use and opportunities provided by UAV imagery for field-based crop phenotyping. They reported that the 88.5% of the surveyed literature about UAV-based phenotyping was published in the last 5 years, which confirms that UAVs have become an important tool in field phenotyping. They also discussed that there is still potential for wider applications, and although automated characterization of flowering dynamics at the field-scale is essential for evaluation of the breeding process, none reference for monitoring flowering was reported, which shows the need for the development of accurate UAV image analysis procedures for flowering characterization.

One of the challenges for the timely generation of accurate information from 3D data from UAV platforms for field-based phenotyping programs is the implementation of robust and automatic image analyses to avoid errors due to subjective manual tasks [26, 29]. Object-based image analysis (OBIA) has showed its efficiency and accuracy for the classification and extraction of information from high spatial resolution imagery [37]. The elemental analysis units of OBIA are not pixels but objects, which are groups of adjacent pixels with homogenous spectral values. In summary, OBIA combines the spectral, topological, and contextual information of these objects to address complicated classification issues. OBIA has addressed the significant challenge of automating image processing in agricultural [18, 38-41], grassland [42, 43], and forest scenarios [44, 45]. OBIA is also being applied to non-image-based sources such as LiDAR point clouds [46, 47], and UAV-derived photogrammetric point clouds [24].

As a part of a broader research project, the work presented herein is a continuation of [24] in which UAV photogrammetric point clouds were automatically created to isolate individual almond trees and quantify their geometrical properties (tree height, length, width, projected area and volume). Once the workflow had been designed and duly validated by Torres-Sánchez et al. [24], the next step was to focus on testing its ability to efficiently phenotype almond varieties in two breeding trials during a complete growing cycle. Therefore, one of the objectives of this investigation was to demonstrate the suitability of a previously reported workflow for almond phenotyping that was not published before. A new methodology, consisting of the use of colors from the point cloud to test the capacity for semi-automatic flowering detection, was also developed. Our work was evaluated by comparing the previous cited procedures with on-ground measurements in two almond fields included in a breeding program through the following specific objectives: (i) automatic detection of the phenotypic differences among almond varieties; (ii) the creation of ranking varieties using different geometrical tree crown properties; (iii) the creation of a flowering calendar, the estimation of flower density, and the comparison between the estimated and observed flowering calendar and dynamics; and (iv) studying the relationship between tree yield and crown volume. To the best of our knowledge, there have been no previous reports about the monitoring of all tree flowering dynamics and flower density of a crop at the field scale using UAV-imagery and remote sensing techniques. The impact of this research is also supported by the use of color information from photogrammetric 3D point clouds using a low-cost RGB sensor on-board an UAV for the timely, accurate, and semi-automatic detection of the flowering cycle. Our main objective was to show that the whole technological and methodological protocol involved is applicable for successful phenotyping of a set of almond varieties in the framework of an almond breeding program.

## Materials and methods

### Study area

**Fig. 1:**
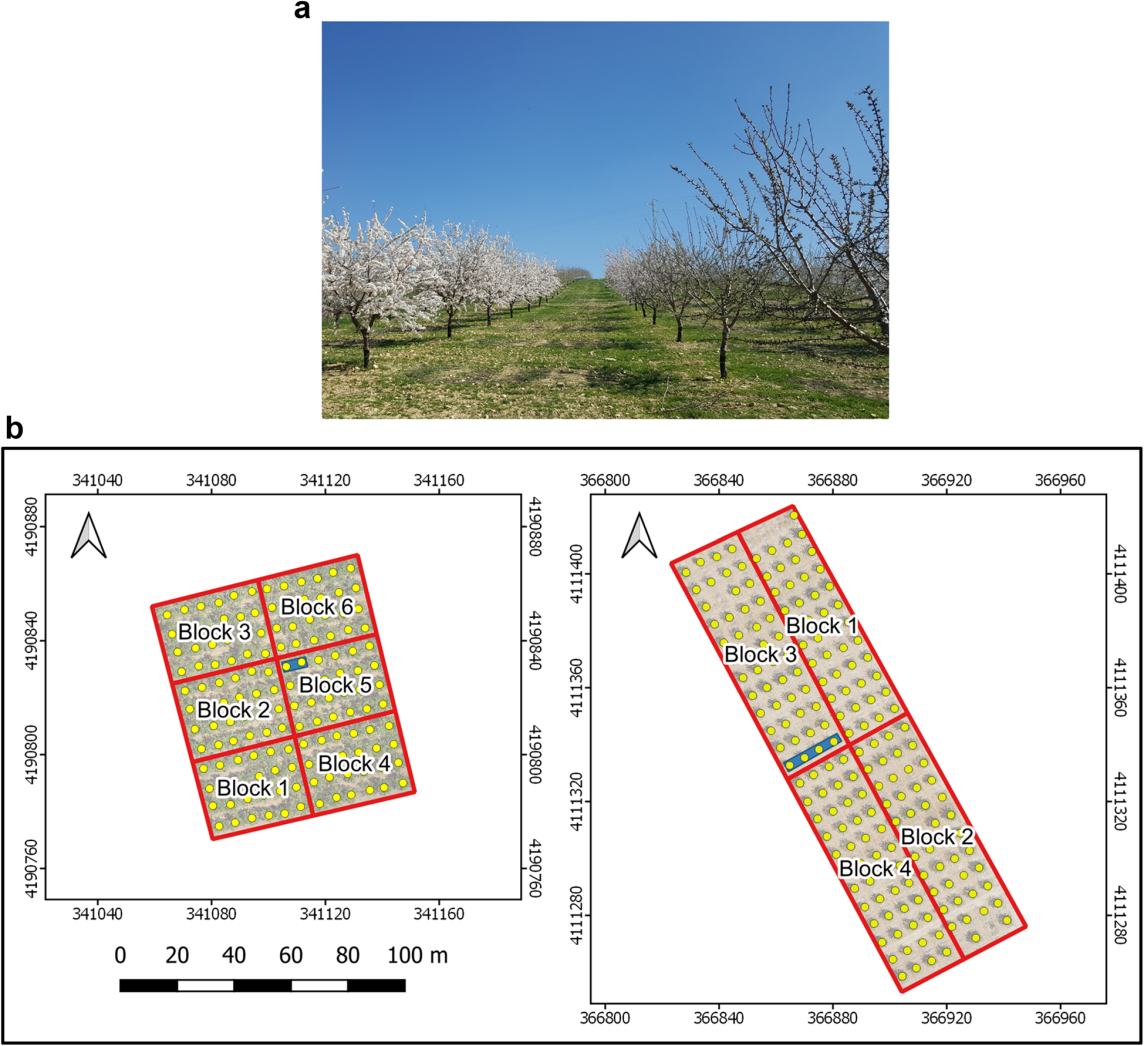
Study area: **a** General view on 2 March, 2017 showing the differences of flowering among almond varieties in the same or contiguous rows; **b** maps of fields 1 and 2, respectively, the blue rectangles depict the grouping of trees belonging to the same variety

The experiment was carried out in two experimental almond orchards (Fig. 1b) located in Andalusia (Southern Spain) which were included in a wide almond breeding program created with the aim of studying the flowering date and the adaptation of almond varieties to different conditions in the Andalusian region. The orchards had tree spacings of 6 × 7 m and were drip-irrigated with a deficit irrigation strategy. Trees were trained in open vase, one the most common and suitable training systems for almond orchard typology.

Field 1 (341,106 O; 4,190,820 N in ETRS89 UTM 30 N) had an area of 0.63 ha with very deep silt loam soil, and it was almost totally flat. The trees were planted in 2000 with a total of 12 Spanish, Italian, and French varieties: Antoñeta (An), Cambra (Ca), Cristomorto (Cr), Ferraduel (Fd), Ferragnes (Fg), Glorieta (Gl), Guara (Gu), Lauranne (La), Masbovera (Mb), Marta (Mt), Supernova (Su), and Tuono (Tu). The field was arranged in a randomized design with six blocks where there were two adjacent trees of every variety, i.e., two trees per elementary plot. This resulted in 142 trees, due to there being two missing trees. Field 2 was on a hillside with a slope of 10% with loam soil, and an area of 0.82 ha (366,883 O; 4,111,348 N in ETRS89 UTM 30 N). The trees were planted in 2012. This field had the same six almond varieties as in field 1, plus six different varieties: An, Belona (Be), Constanti (Co), Fd, Fg, Gu, La, Marinada (Ma), Soleta (So), Tarraco (Ta), Tu, and Vairo (Va). The experimental setup consisted of four blocks in a randomized design with four adjacent trees of every variety. Due to there being seven missing trees, this field had 185 trees.

**Fig. 2:**
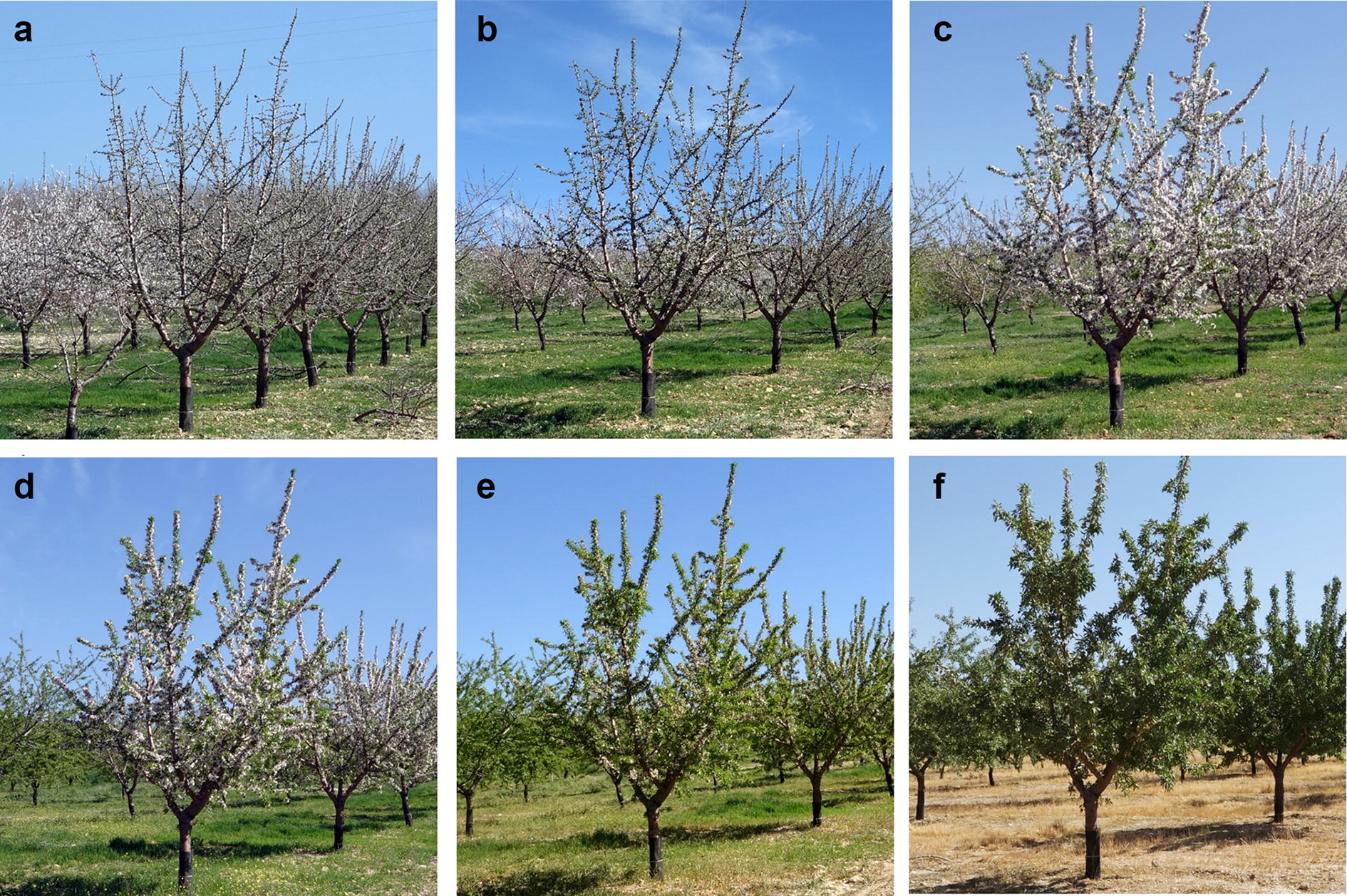
Set of on-ground images showing the flowering and canopy dynamics of the same almond tree in field 2 during the different flight and field measurement dates in March and June: **a** 03-02-2017; **b** 03-08-2017; **c** 03-15-2017; **d** 03-20-2017; **e** 03-28-2017; **f** 06-21-2017

With the objective of monitoring the almond trees, a set of UAV flights and on-ground measurements were carried out several times during the 2017 growing period, allowing temporal information about the different varieties to be gathered. Table 1 shows the dates of data acquisition and the corresponding crop stage for every field, and Fig. 1a illustrates the differences in flowering among varieties in field 2, whereas Fig. 2 shows the flowering and canopy evolution of one of the trees on the different dates. It can be seen that the almond tree located to the left of the main tree displayed in Fig. 2a presented more intensive flowering, showing a different blooming stage typical of breeding programs when a collection of different varieties was tested (Fig. 1a). There were more acquisition data dates in field 2 due to its almond varieties showing a wider flowering calendar range.

**Table 1:** Date and growth stage of every acquisition data day during 2017

Stage	Month	Day
Flowering and leaf development	March	Field 1: 1, 9, 16 Field 2: 2, 8, 15, 20, 28
Crown fully developed and with mature nuts	June	Field 1: 22 Field 2: 21
Post-harvest	September	Field 1: 25 Field 2: 26

### Three-dimensional point cloud generation

The 3D point clouds used for the monitoring of the geometric characteristics of every almond tree were created by applying photogrammetric and computer vision techniques to the images acquired with an UAV. The imagery was taken with a low-cost, commercial, off-the-shelf camera (Olympus PEN E-PM1, Olympus Corporation, Tokyo, Japan) equipped with a 14–42 mm zoom lens that was fixed to a 14 mm focal length for this study. The camera was mounted in the UAV, a quadcopter model (MD4-1000, microdrones GmbH, Siegen, Germany), facing downward for nadir capture. The flight routes were designed with forward and side laps of 93% and 60%, respectively, and the flight altitude was 50 m, which led to a ground sampling distance of 15.3 mm. According to previous investigations, this configuration is the optimum one to achieve the 3D reconstruction of woody crops [48]. All the flights were done on sunny days with low wind speeds (< 6 km h^−1^).

**Fig. 3:**
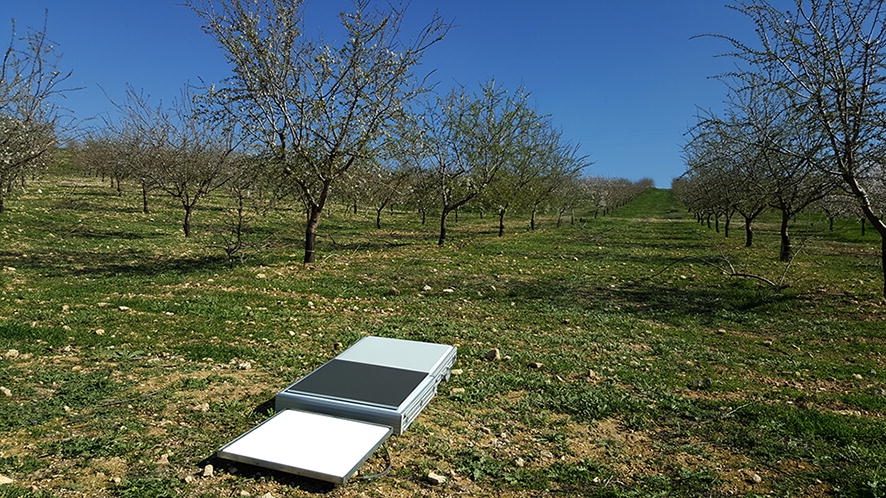
White, black, and grey reference panels in field 2 on 2 March 2017

A white Spectralon^®^ panel (Labsphere Inc., North Sutton, NH, USA), a grey panel, and a black panel (SphereOptics GmbH, Uhldingen, Germany) of 0.45 × 0.45 m were placed in the middle of the fields to take into account the light changes along the different flight dates (Fig. 3). The Spectralon, grey, and black panels had reflectance in the visible range of about 97%, 43%, and 5%, respectively.

The photogrammetric software used for the generation of the 3D point clouds was Agisoft PhotoScan Professional Edition version 1.2.4, build 1874 (Agisoft LLC, St. Petersburg, Russia). The process was fully automatic, with the exception of the manual localization of 5 ground control points taken with a real time kinematic (RTK) GPS linked to a reference station from the GNSS (Global navigation satellite system) network from the Institute for Statistics and Cartography of Andalusia (IECA), Spain. The estimated accuracy of the GNSS-RTK system was 0.02 m in planimetry and 0.03 m in altimetry. The point cloud was saved in “.*las*” format, a public file format for the interchange of 3D point cloud data. More information about the processing parameters of the software can be read in [24].

### Almond tree characterization

The geometric features of every tree, such as the crown projected area, tree height, and crown volume, were derived using the OBIA algorithm that was previously developed for almond trees trained in open vase in [24]. The validation of the algorithm showed that the comparison between tree height estimation and measured tree heights produced an R^2^ of 0.94; and all the accuracy measures for tree crown delineation were over 0.9 in a range from 0 to 1.

As part of a broader research project, the work herein presented is a continuation of that research, since once the workflow was designed and duly validated, the next step was primarily focused on testing its ability to efficiently phenotype almond varieties in breeding programs (“OBIA algorithm for crown architecture” section). The next step was to create a new procedure consisting of the use of color from the point cloud to monitor the flowering calendar (“Flowering detection” section). Both objectives aimed to generate the required information to quantify the canopy geometry at different phenological stages and to monitor the flowering dynamics for phenotyping each tree variety.

#### OBIA algorithm for crown architecture

As stated in [24], the OBIA algorithm for the characterization of every almond tree size and geometry does not require user intervention and can be divided into the following successive, automatically executed steps: (i) digital terrain model (DTM) generation; (ii) tree crown delineation; (iii) point cloud slicing; and (iv) almond tree 3D characterization. The only inputs in these steps were the point cloud and a shapefile delimiting the studied field. The algorithm output was a vector file that was ready to be used in any geographic information system and included the crown limits of every tree and, as associated information, its crown volume, maximum height, projected area, length, and width. This information could be also exported as an ASCII table including the previous information plus the central coordinates for each tree. The programming language Cognition Network in eCognition Developer 9 software (Trimble GeoSpatial, Munich, Germany) was used to develop the OBIA algorithm.

#### Flowering detection

A new methodology based on the use of point colors to test the capacity for flowering detection was developed. The basic idea was to use the color of the almond flowers for semi-automatic flowering estimation by comparing the brightness of the flowers with the brightness of the reference panels. Some authors have reported the use of a single color feature for flower detection in images. For example, Carl et al. [49] used information from the blue band, and Richardson et al. [50] used the relative brightness of the red channel. CloudCompare 2.8 (EDF R&D and Telecom ParisTech, Grenoble, France) software was used for point cloud processing in this section, and the analysis was only applied to the point clouds from March, since there were no flowers in June or September. In a first step, the brightness levels of all the points in the point clouds were calculated using Eq. (1), where R, G, and B were the digital numbers for the red, green, and blue bands of the points, respectively:

1 $$Brightness=\frac{R+G+B}{3}.$$

Then, the average brightness of the points corresponding to grey and white reference panels was calculated, which allowed the different light conditions on the different flight dates to be taken into account. The points with brightness levels equal to or higher than the panel average brightness and with a height over the DTM higher than 1 m were classified as flower points. As the limits of each tree were known based on the OBIA algorithm involved in “OBIA algorithm for crown architecture” section, the number of flowers inside each almond crown was extracted. The flower density (number of flowers m^−3^) was calculated by dividing the number of points identified as flowers inside a tree crown by its volume.

### Data analysis

All of the following statistical analyses were carried out using JMP software (SAS, Cary, NC, USA).

#### Flowering detection

The dates of the beginning and end of blooming and full blooming for each variety were registered according the BBCH Monograph (2001) through visits twice a week to the experimental fields as follows: (i) beginning of flowering (about 5% of flowers open); (ii) full flowering (50% of flowers open); (iii) end of full flowering (90% of flowers open and first petals fall); (iv) end of flowering (5% of flowers and 95% of petals fallen); and (v) end of flowering (all petals fallen). The goodness evaluation of the flowering estimations was done by comparing the temporal trend of the flower density with the flowering evolution as observed on every field.

#### Ranking varieties

One of the main interests of phenotyping studies is to elaborate ranking varieties for a concurrent comparison of the target traits for the different varieties [51, 52]. The OBIA estimations of tree height and crown volume were used to create a ranking of varieties’ sizes through a comparison of means with the Tukey HSD test (*p* < 0.05). These rankings were created in both fields by using every almond tree height measured using a clinometer in September the same day of the flight, which allowed the height rankings from the OBIA algorithm and the corresponding field data to be compared.

#### Estimated yield and volume

Yield data were collected in both fields at harvest time, the first and second fortnights of August, for fields 1 and 2, respectively. The harvest was done mechanically using a tree shaker. The harvest weight could not be recorded on an individual basis due to labor costs and time constraints. Consequently, the raw harvests of adjacent trees of the same variety were grouped for weighing. Taking into account the number of blocks in every field and the fact that the trees of the same variety were grouped in every block, there were six yield values per variety in field 1, whereas there were four yield values per variety in field 2. The yield values in field 1 averaged two trees, while in field 2, they averaged four trees. The relation between the average tree volume obtained by the OBIA algorithm and the average yield was evaluated by calculating the determination coefficient (R^2^) and the significance of the linear fit for each date, field, and variety.

## Results and discussion

### Flowering detection

**Fig. 4:**
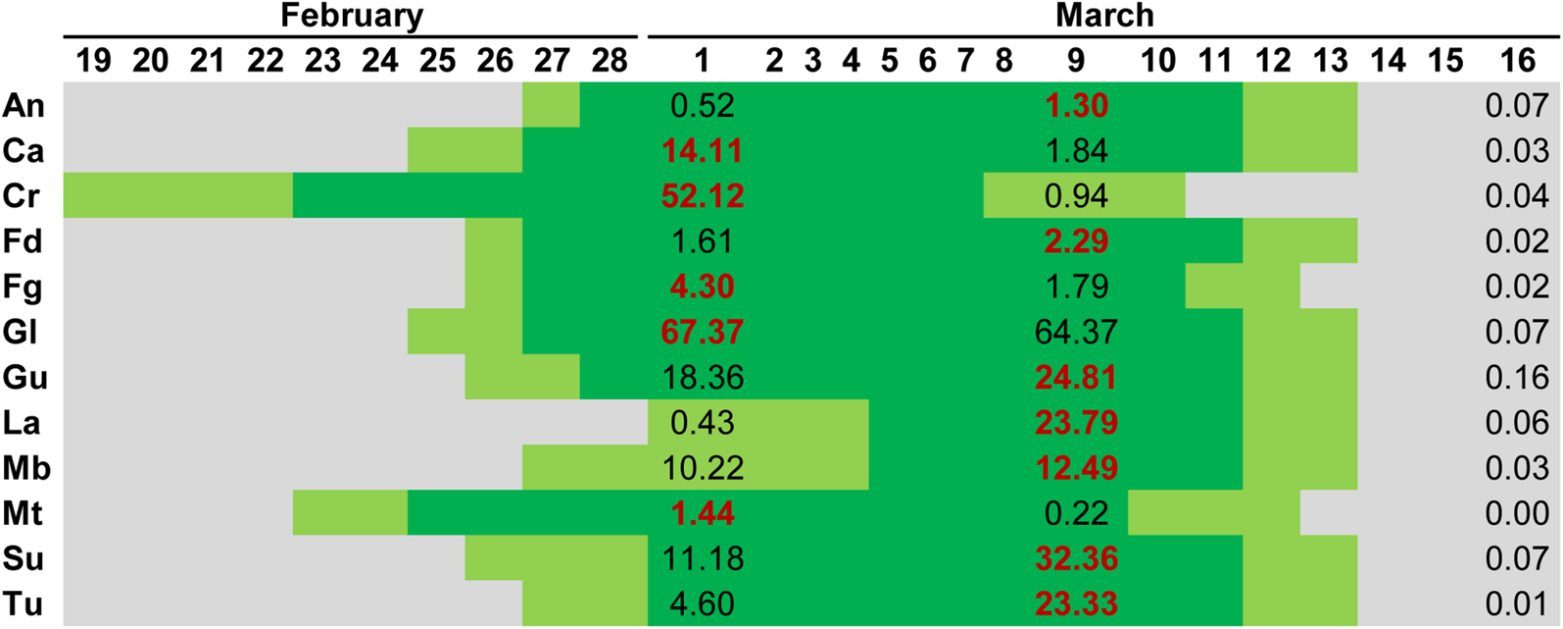
Blooming calendar for field 1. Dark green days indicate full blooming period, and light green days indicate the beginning and ending date of blooming. Numeric values report the averaged flower density (number of flowers m^−3^) for each variety, red bold values are the maximum flower density for each variety

**Fig. 5:**
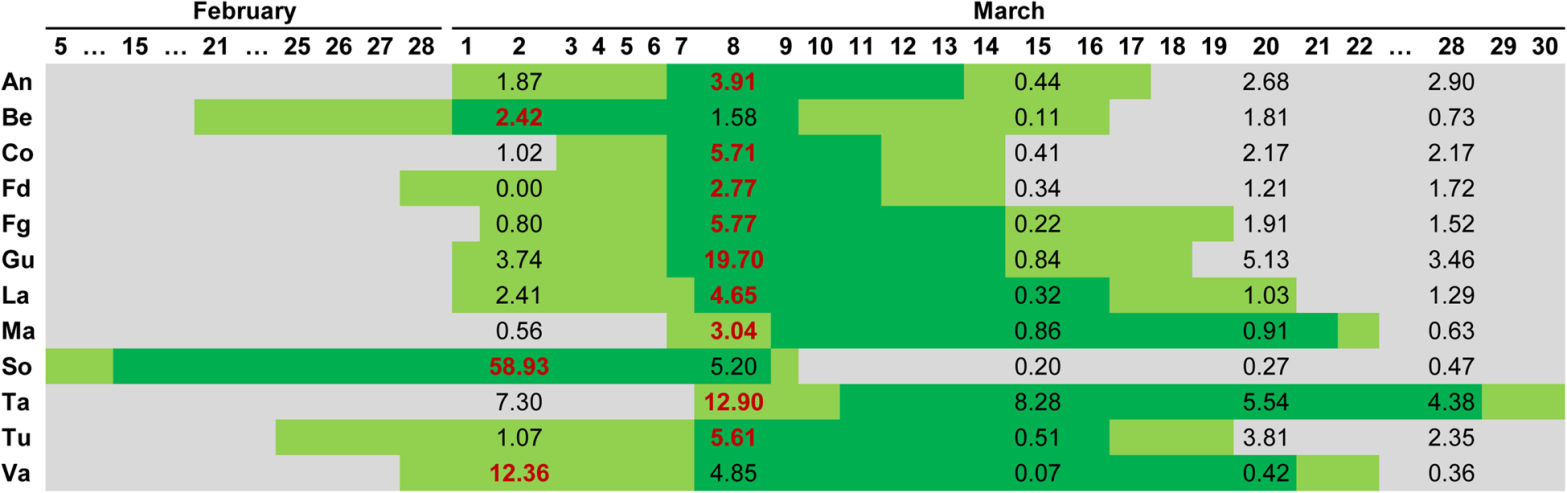
Blooming calendar for field 2. Dark green days indicate full blooming period, and light green days indicate the beginning and ending date of blooming. Numeric values report the averaged flower density (number of flowers m^−3^) for each variety, red bold values are the maximum flower density for each variety

Figures 4 and 5 show the high level of agreement between the flower density (number of flowers m^−3^) estimations and the blooming calendar observed on both fields. Although the average brightness of the points corresponding to grey and white reference panels was calculated, the results presented and discussed in this section were produced using the grey panel as the brightness reference, since it better covered the white and purple range of almond flower colors that different varieties can show, as assessed by Gülcan [13]. In addition, the flower density values estimated using the white panel did not adjust to the blooming calendar as its brightness values were too high and its use as a threshold resulted in a scarce number of points being classified as flowers (data not shown). Underwood et al. [53] applied a fixed color threshold to images for flower detection in almond trees by using a mobile robotic ground scanning system; however, they admitted the need to use a more advanced method that would take into account variations in illumination. For this reason, a variable threshold based on the reflectance of the grey reference panel was used in this research, which allowed the generation of more accurate results.

The maximum values of flower density for all the varieties agreed with the full blooming period observed in field 1 and all flower density values obtained by the UAV imagery on 16 March were near zero when the blooming period had finished (Fig. 4). In field 2, the maximum flower density in 9 out of 12 varieties was coincident with the full blooming period (see 8 March, Fig. 5). In two of the varieties with no agreement (Ma and Ta), the differences between the maximum estimated flowering and full bloom observed were only 1 and 3 days, respectively. In the other variety with no agreement (Va), the difference was 6 days. These are considered low differences taking into account that the blooming period takes around 20 days for those varieties. In field 2, all varieties showed very low flower density values on 15 March, followed by a small increase in this value on 20 March. This can be explained by the intense rainfall registered on 14 March that could have provoked the fall of petals. Although, taking into account the characteristic continuous blooming of almonds, some trees could have bloomed again after this rainfall because their flowering period was not over, indicating the suitability of the procedure for continuous monitoring of flowering. Underwood et al. [53] also reported analogous flowering dynamics on a daily time-scale since the number of flowers increased due to continuous blooming and decreased due to falling petals. Four out of the six common varieties in both fields (Fd, Gu, La, Tu) showed similar flowering tends, i.e., maximal flower densities in the UAV imagery taken on the 8th and 9th of March.

Hand counts for flower density are difficult to obtain in a single day for large numbers of almond trees, because each tree contains upwards of 10,000 flowers [53]. For this reason, traditional flowering studies in almonds have been based on careful data acquisition from two branches (1 year old shoots and spurs) per tree selected at random on a subset of trees inside the orchard and by counting flower bud density (bud cm^−1^) in a length of 1 m [54]. The relatively small number of individual sample trees used in former studies on the flower density is a constraint compared to the information obtained per every tree by using the point clouds and the fully automatic OBIA algorithm. Therefore, one of the main results of the approach proposed in our study is that it allows the estimation of flower density of all trees in an orchard in a very efficient way. However, as previously reported by Underwood et al. [53], it was not possible to evaluate the absolute accuracy of the flower estimations due to the lack of direct data reporting the total number of flowers in the almond tree crowns. Additionally, this information about flowering density and the creation of flowering dynamics or calendars is of crucial importance for almond phenotyping due to three main reasons: (i) it can be performed on a single day per sampling period by using a low-cost camera on-board a UAV platform; (ii) one of the most important objectives in almond breeding is the creation of ranking varieties for late-blooming that are able to avoid late-winter/early-spring frosts which affect almond production because of its very early flowering season [55]; and (iii) the flowering density is related to the productive potential and consequently, to the commercial value of a new variety [7]. It can be also stated that when frost risks are low, as happens in California, the breeders tend to have a prevalence of cultivars with low or medium flower density, probably to reduce the problems of fruit quality decrease related to a higher percentage of double kernels [56]. In contrast, in regions with high frost risks in early spring (e.g., Spain), a high flower bud density has been considered a positive trait for cultivar evaluation as it compensates for flower damage and ensures an acceptable crop yield [56].

Previous approaches analyzed UAV imagery in woody scenarios to map the flowering of the invasive plant *Acacia longifolia* using random forest to test a biocontrol agent, or *Robinia pseudoacacia* to quantify the habitat potential for honeybees (*Apis mellifera*) [49, 57], or in herbaceous crops such as oilseed rape (*Brassica napus*) to determine the flower fraction and flower number [58, 59]. However, to our knowledge, this is the first time that the flowering density of a crop has been detected using color information from photogrammetric point clouds. Furthermore, this is the first time that the entire blooming dynamics of a woody crop have been monitored using an UAV with relevant implications in the breeding program, since a high variability in flowering time and flower density could be accurately monitored.

The advantage of using a low-cost sensor-based UAV for crop phenotyping was shown in our study, as coverage of the whole field could be achieved in 7 and 10 min for fields 1 and 2, respectively. Using a mobile robotic ground scanning system, Underwood et al. [53] scanned both sides of the almond rows in the morning and afternoon, taking considerably more time for all sampling trees. They also discussed that the commercial implementation of their sensing strategy would require lower cost platforms (e.g., a bolt-on sensor located in the farm vehicles) than the custom-made robot they used.

### Ranking varieties

#### Ranking varieties for height

**Fig. 6:**
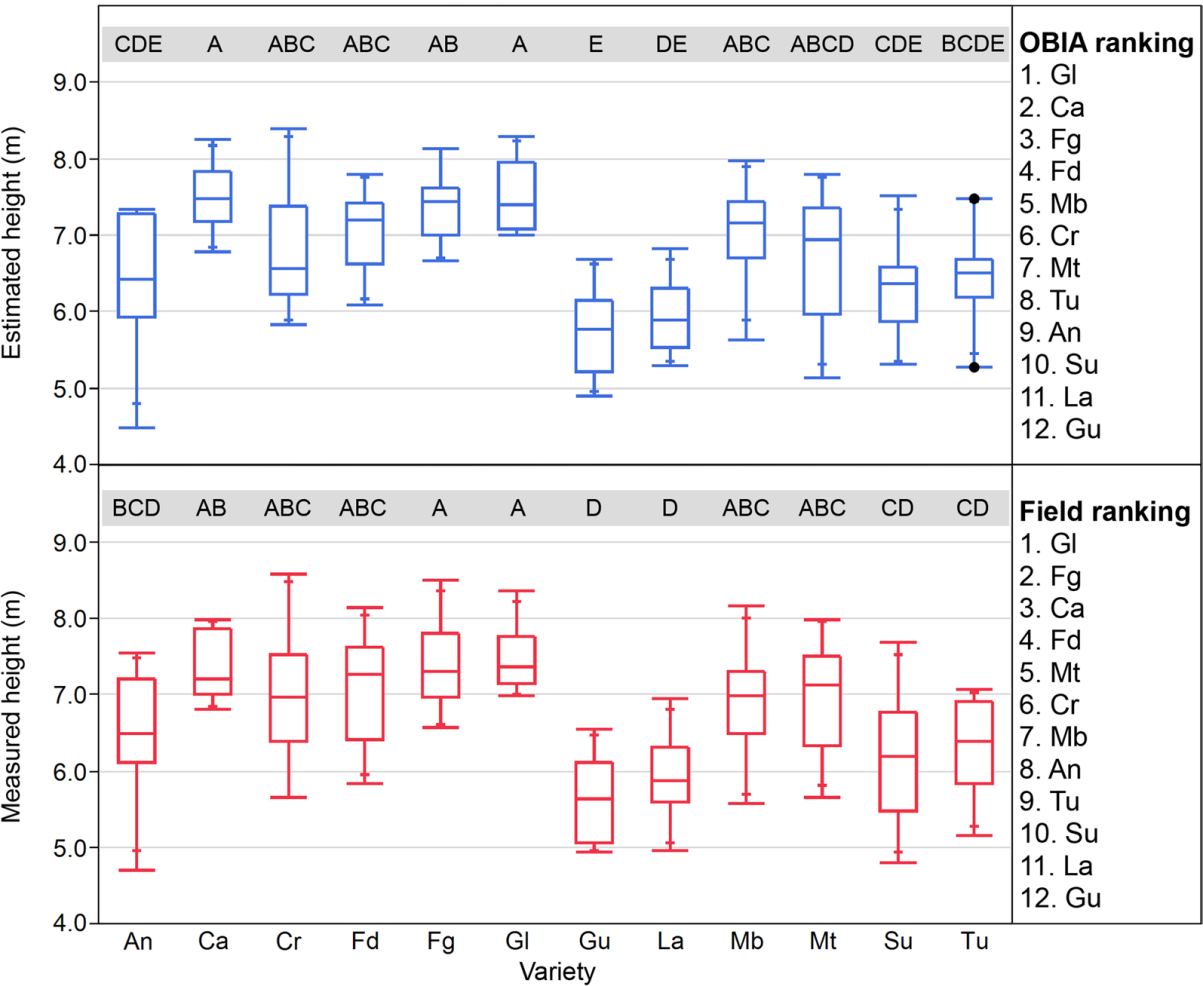
Boxplots for the measured and estimated height of the almond trees in field 1 by variety, where the points indicate outliers. Different letters indicate significant differences (Tukey HSD test, p < 0.05)

**Fig. 7:**
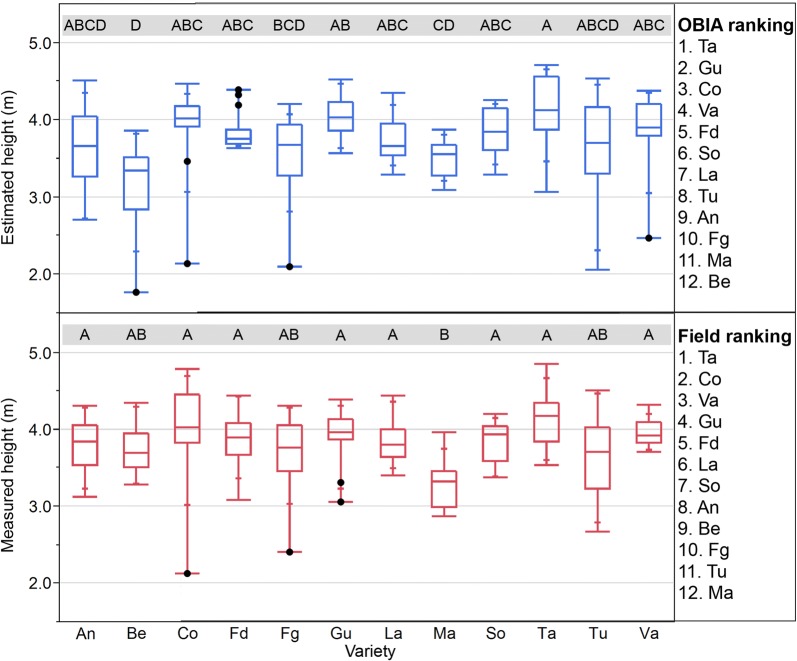
Boxplots for the measured and estimated height of the almond trees in field 2 by variety, where the points indicate outliers. Different letters indicate significant differences (Tukey HSD test, p < 0.05)

As stated previously, the creation of ranking varieties is one of the most used tools in breeding programs to select the most preferred varieties according to the trait under study. Therefore, considering that the differences in height among the varieties were consistent for UAV-estimated and field-measured heights in fields 1 and 2, two ranking varieties for height were constructed, and a comparison between both rankings was done for both kinds of measurements (Figs. 6, 7). The selection of the four tallest varieties in field 1 was coincident for the OBIA and the traditional methodologies (Gl, Ca, Fg, Fd; Fig. 6), although these four varieties were not exactly in the same order. Concerning the detection of the four smallest varieties, there was full agreement in variety selection and its ranking position for three of them (Su, La, Gu). In field 2 (Fig. 7), the differences in height were lower than in field 1, probably because the trees were only 5 years old, and the phenotypic differences in tree height were less developed than in field 1. The statistical study of the height data showed less significant differences for the on-ground data than for the UAV data. However, there was coincidence in the selection of the tallest five varieties (Ta, Gu, Co, Va, Fd), although not in its position. The agreement between manual and OBIA ranking was minor in the selection of the three smallest varieties, with the coincidence of two of them (Fg, Ma).

The workflow proposed in this work was able to detect the phenotypic variability in height in the experiment and to generate ranking varieties with a high degree of similarity to the one created from traditional on-ground measured data in research fields with trees of different ages. The main advantage of the proposed workflow in comparison with traditional manual measurements is its efficiency. As reported in [24], orchards, like field 1, can be analyzed in 1 day by a single person (including UAV flight, 3D model generation, and analysis) allowing the extraction of accurate information of crucial relevance in breeding programs, such as height, area, position, volume, crown length and width, and flower density, for every tree.

#### Ranking varieties for volume

**Fig. 8:**
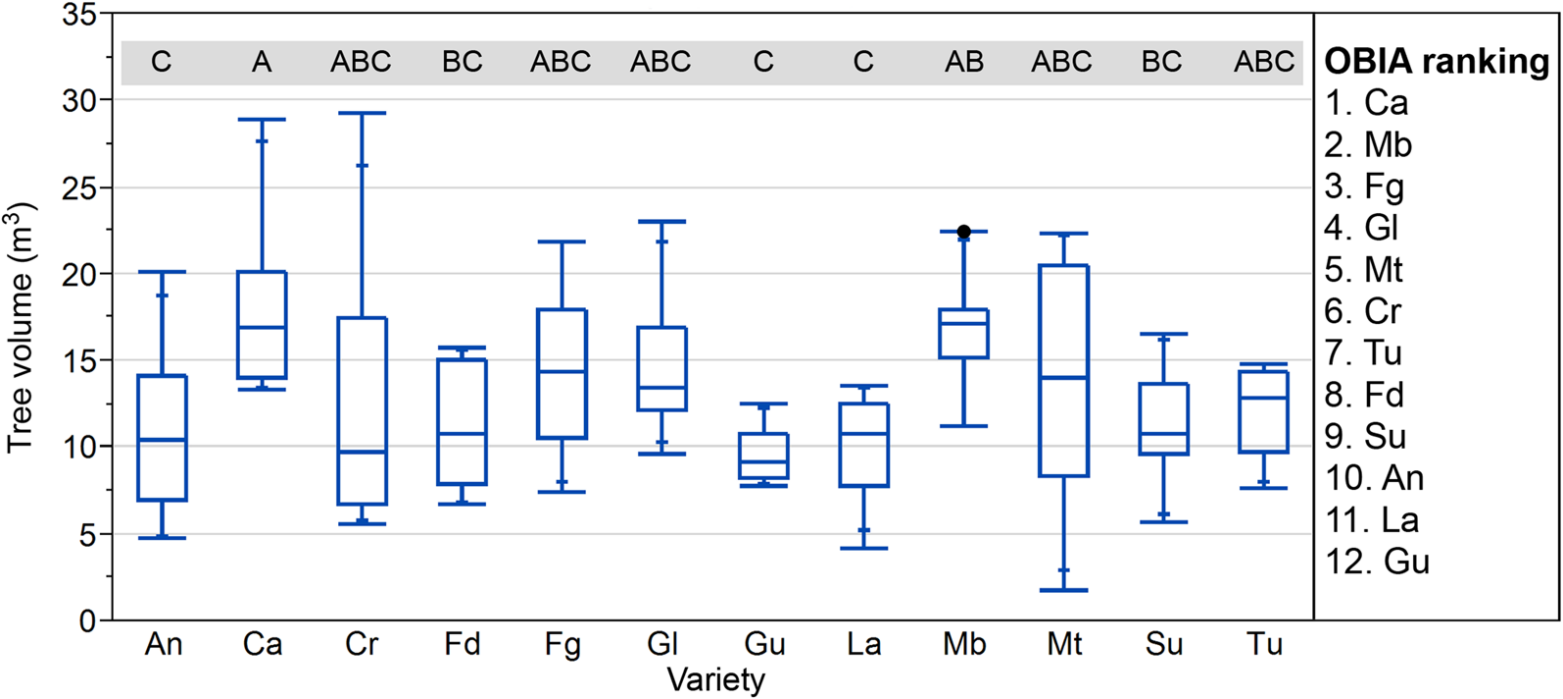
Boxplots for the estimated crown volume of the almond trees in field 1 by variety, where the points indicate outliers. Different letters indicate significant differences (Tukey HSD test, p < 0.05)

**Fig. 9:**
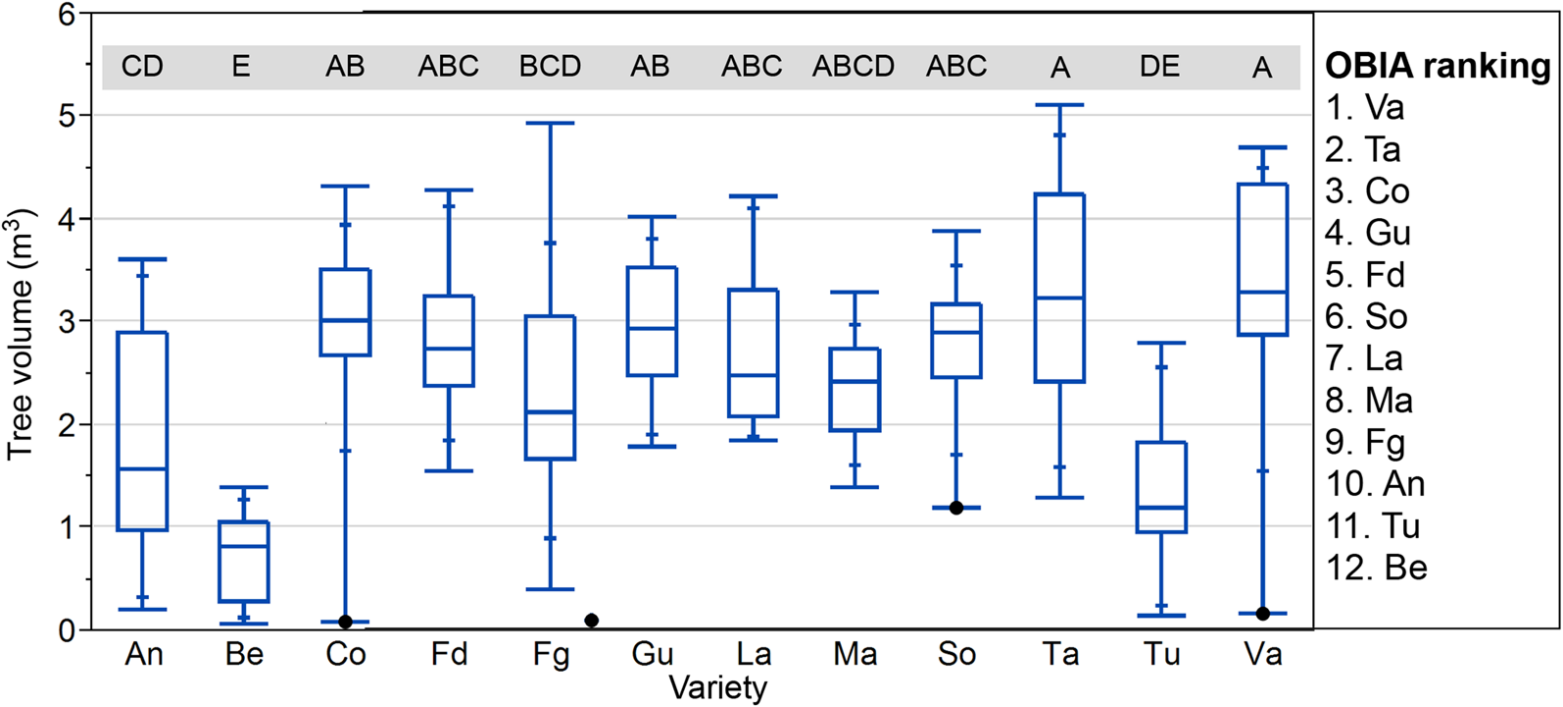
Boxplots for the estimated crown volume of the almond trees in field 2 by variety, where the points indicate outliers. Different letters indicate significant differences (Tukey HSD test, p < 0.05)

In addition to the ranking varieties based on tree height, the output of the OBIA algorithm allowed the creation of ranking varieties based on the differences in tree volume for every variety. Figures 8, 9 show the differences in fields 1 and 2. There were differences in the ranking order of some varieties depending on the studied variable. For instance, Fd was the fourth tallest variety in field 1 (Fig. 6), but it was in eighth position in the volume ranking (Fig. 8). In field 2, Ma was the penultimate variety in relation to height, but it was in eighth position in the volume ranking (Fig. 9). The differences in the order of ranking illustrate the differences in canopy architecture among varieties, showing that the genetic factors controlling phenotypic traits are highly conditioned by the environmental conditions of each screening site; that is, a variety can show a different growing habit in different years and locations [60].

The interpretation of information derived from the geometric features is complex. For example, if a variety has an upright growing habit [13], but it does not have a voluminous crown and its foliar density is low, it could mean that it has weak branching intensity development, which is related to a lower flower bud density. This potential low flower density could be also relevant to assess the possibility of enduring a late frost during bloom, because the presence of a high number of flowers increases their ratio of survival after a frost, consequently affecting the productivity parameters [54]. The information about the geometric crown features of the different varieties can be also useful to select the best planting pattern (e.g., a variety with a high volume crown needs a higher tree spacing) or to choose between varieties according to the required pruning system, which influences the production costs. Other specific multi-criteria to rank varieties based on tree features generated by the OBIA algorithm could be created, as has been done with tree height and volume (Additional file 1).

### Estimated yield and volume

The study of the temporal relationship between the estimated volume and the raw harvest weight showed that some varieties have significant R^2^ values (R^2^ > 0.7 with *p* < 0.05) (Tables 2, 3). In field 1, the An and Su varieties showed a significant linear fit between the two variables in March during the flowering leaf development stage. The crown volume and yield were well related for Cr in June when the almond trees were mature. The Ca and La varieties also had significant correlations, although these varieties had an R^2^ value of < 0.7. In field 2, four varieties (Be, Fd, La, and Va) showed highly significant R^2^ values in March, while Gu and So had good relations in both March and June. All of these coefficients suggest that the canopy volume is related to the yield, although the linear relationship is different for each variety. This trend indicates that the tree canopy volume during flowering extracted from UAV imagery may be a good indicator of yield estimation, although a wider sample size covering more data from harvest and years is needed to produce consistent conclusions.

**Table 2:** Crown volume relationship with yield for field 1 (six samples per variety, and each sample is the mean of two trees)

Varieties	01 March	09 March	16 March	22 June
An	0.76**	0.43	0.40	0.22
Ca	0.42	0.05	0.56*	0.59*
Cr	0.44	0.10	0.68**	0.80**
Fd	0.00	0.09	0.21	0.02
Fg	0.01	0.30	0.01	0.01
Gl	0.17	0.12	0.11	0.02
Gu	0.13	0.21	0.18	0.12
La	0.18	0.65*	0.56*	0.67**
Mb	0.07	0.02	0.03	0.07
Mt	0.28	0.38	0.26	0.11
Su	0.73**	0.65*	0.40	0.39
Tu	0.02	0.00	0.14	0.01

Italic values indicate significant relationships with R^2^ > 0.7

*ns* not significant

^**^p < 0.05, *p < 0.10

**Table 3:** Crown volume relationship with yield for field 2 (four samples per variety, and each sample is the mean of four trees)

Varieties	02 March	08 March	15 March	20 March	28 March	21 June
An	0.04	0.05	0.03	0.08	0.34	0.25
Be	0.39	0.84*	0.40	0.23	0.12	0.37
Co	0.66	0.71	0.60	0.64	0.39	0.42
Fd	0.88*	0.13	0.26	0.97**	0.93**	0.40
Fg	0.00	0.47	0.78	0.66	0.75	0.74
Gu	0.33	0.85*	0.98**	0.92**	0.89*	0.95**
La	0.83*	0.99**	0.99**	1.00**	0.98**	0.77
Ma	0.33	0.74	0.63	0.41	0.61	0.79
So	0.48	0.32	0.84*	0.92**	0.74	0.89*
Ta	0.10	0.38	0.01	0.06	0.16	0.06
Tu	x	X	X	X	X	X
Va	0.96**	0.83*	0.92**	0.71	0.52	0.06

Italic values indicate significant relationships with R^2^ > 0.7

*ns* not significant, *X* no data

^**^p < 0.05, *p < 0.10

Grouping the data from the different varieties for a single linear relationship between volume and yield did not work out, and the R^2^ values obtained were below 0.20 in all dates for both fields. This is in agreement with [53] who lumped the yield and volume obtained using an on-ground LiDAR system together and obtained a linear relationship for a set of almond varieties of R^2^ < 0.39. In contrast, they reached a R^2^ value of > 0.7 for all four studied varieties for the linear fit between the tree estimated volume and yield during the flowering stage. They extrapolated their data for yield prediction in the whole orchard, although they did not validate their results. According to Hill et al. [4], other canopy features, such as the size of almond trees considering the cross-sectional area and assuming a circular section, can be used to estimate the almond yield. They also listed a set of recommended measures required for a good estimation of yield. In [61], an R^2^ value of 0.8 was achieved between the yield and tree crown volume of orange trees using an ultrasonic sensor, although when the linear fit was extrapolated for yield prediction, they obtained an R^2^ value of < 0.42 in the validation.

The detected density of flowers could also be tested for yield prediction. However, there are external factors that can distort its relation with the yield, such as the availability of pollinators, the amount of fruits that a tree can bare [62], and intense rains or late frosts that can harm the flowers, having a negative influence on the activity of pollinating insects [55]. Furthermore, Underwood et al. [53] reported that the absence of relationship between detected flowers and yield could be reasonable due to the large number of factors influencing pollination and fruit set. They also suggested that adding flower densities to the modeling of yield based on almond volume does not provide additional benefit.

Future research will focus on the acquisition of more detailed yield data to improve the linear fit between the detected crown volume and the yield, which could help to elucidate the date with the best R^2^ values. All of this information could be used to generate yield prediction models for the studied almond varieties that would be useful to ease orchard management, plan the harvest labor, or to prepare post-harvest tasks such as drying and storage. Yield prediction could also be used to apply variable rate fertilization or other inputs depending on the predicted yield, which would result in more efficient management of an orchard, as reported in an apple orchard by [63].

## Conclusions

Two almond phenotyping trials with trees of different ages were evaluated throughout a complete growing cycle using an approach based on two steps: (i) the generation of photogrammetric point clouds from images acquired with a low-cost camera on-board a UAV; and (ii) analysis of the point clouds using a fully automatic OBIA algorithm. The analysis allowed the extraction and quantification of the following almond features that are of paramount importance in almond phenotyping: flowering density, tree height, and even, crown volume for every tree and variety. The temporal evolution of the flower density estimations from the UAV imagery showed a high level of agreement with the blooming calendar observed from field measurements on both fields. Data from the OBIA analysis of the 3D point clouds were useful for detecting the phenotypic variability in the almond orchards, and allowed the creation of variety rankings with a high degree of similarity to the one created from manually measured data. Some of the almond varieties were found to show a significant linear fit between crown volume and yield. To our knowledge, this is the first time that photogrammetric point clouds have been used for the detection of flowering in a crop. Furthermore, this is the first time that the entire blooming period of a woody crop has been monitored and that consistent variety rankings are created using a UAV-imagery.

From the results achieved in this research work, it can be said that the suitability for almond phenotyping of the proposed UAV-based workflow has been demonstrated, which has not been reported in any previously published research. The main advantage of this workflow in comparison with traditional and laborious approaches is the ability to generate a large amount of useful data in a timely manner with reduced field work in an efficient, non-destructive, and inexpensive way. This technological and methodological tool could be adapted to provide technical support to promote commercially feasible applications of UAV in crop phenotyping of other woody crops (e.g., apple, peach, olive) for other researchers or breeding companies. In addition, the mapped geometric tree features can be used as a baseline tool for site-specific almond tree management according to the crown architecture parameters in case of a fungal foliar disease (e.g., red leaf blotch of almond, *Polystigma amygdalinum*) or other negative canopy circumstance occurring during the breeding program.

Future investigations could be focused on the development of comparative analyses of almond ranking varieties to test their growing habits, flowering dates, and yields over multiple locations and years to select the best performing and stable genotypes. Considering that our work was on almond trees trained as single trunk open vase (i.e., discontinuous canopy), next work could focus on continuous canopy (narrow hedgerow), since breeders may need to study and produce cultivars that are also suitable for use in this type of cropping system.

## Supplementary information

**Additional file 1.** Video overview of field almond tree phenotyping using RGB-UAV-based platform.

## Data Availability

The datasets used and/or analysed during the current study are available from the corresponding author on reasonable request.
